# Virtual Reality Interventions of Daily Versus Weekly Data Collection in Patient-Reported Outcomes Among Adults With Cancer: Pilot Survey Study

**DOI:** 10.2196/73506

**Published:** 2025-07-29

**Authors:** Matthew Browning, Olivia McAnirlin, Fu Li, Jeffrey Bertrand, Denise Davis, Kapil Madathil, George Fredric Mau, Teny Henry Gomez

**Affiliations:** 1 Virtual Reality and Nature Lab, Department of Parks, Recreation and Tourism Management, Clemson University, 515 Calhoun Drive, Clemson, SC, 29631, United States, 1 864 656 3400; 2 Department of Industrial Engineering, Clemson University, Clemson, SC, United States; 3 Department of Integrated Information Technology, Molinaroli College of Engineering and Computing, University of South Carolina, Columbia, SC, United States; 4 Watermark Counseling, Columbia, SC, United States; 5 Division of Hospice and Palliative Medicine, Department of Internal Medicine, Prisma Health Upstate, University of South Carolina School of Medicine Greenville (USCOM-Greenville), Columbia, SC, United States

**Keywords:** virtual reality, guided imagery, survey administration, pain management, research design

## Abstract

**Background:**

Virtual reality (VR) interventions are increasingly used in health care settings to improve patient-reported outcomes (PROs). PROs are commonly evaluated at weekly intervals with data collected via digital surveys. While weekly assessments have benefits, VR devices enable more frequent in-device data collection. It remains unclear whether PROs collected more frequently provide more information on these interventions than PROs collected more infrequently.

**Objective:**

This pilot study explored differences between daily and weekly PRO data collection in a VR intervention with nature imagery, with and without guided imagery, among patients with cancer.

**Methods:**

Patients with cancer (n=8) were randomly assigned to one of four intervention groups: (1) virtual reality–assisted guided imagery (VRAGI), (2) VR without guided imagery, (3) desktop VR with guided imagery, or (4) desktop VR without guided imagery. Devices were mailed to participants’ homes for 15‐20 minutes of daily use over 3 weeks. Weekly outcomes (pain, anxiety, depression, and well-being) were assessed using items from the Edmonton Symptom Assessment Scale. Daily outcomes were captured via in-device pre-post surveys. Data were analyzed descriptively, using visual trend comparisons to explore patterns.

**Results:**

Of 41 patients who consented, 8 provided complete and usable data. Weekly outcomes showed no consistent trends. In contrast, daily data revealed more nuanced patterns, such as early symptom relief, plateaus, and “double-bottom” effects. The addition of guided imagery did not consistently enhance outcomes beyond VR alone, although the VRAGI condition showed the greatest improvement in well-being. Given the small sample size, these findings should be considered exploratory.

**Conclusions:**

This pilot study suggests that daily PRO data might offer richer insight into intervention effects than weekly assessments. Further research with larger samples is needed to confirm these patterns.

## Introduction

Health care interventions using virtual reality (VR) are often evaluated by testing their efficacy at weekly intervals [1,2]. Data can be collected via digital surveys, paper questionnaires, SMS text messages, or physician notes on medication use and psychological outcomes (ie, pain, anxiety, and depressive symptoms) and compared with these same measures weeks later [3,4]. For example, Pallavicini et al [5] compared stress, anxiety, and depression levels before and after 1 week of VR at-home intervention, and Hernandez et al [6] compared depression, well-being, and quality of life immediately after VR, as well as 3 and 6 months after VR.

Collecting data relatively infrequently (ie, weekly) has benefits. Using weekly push notifications from the REDCap (Research Electronic Data Capture) system, patients can complete HIPAA (Health Insurance Portability and Accountability Act)-compliant surveys with minimal burden [7]. Additionally, patients may be more likely to complete surveys if assessments are spaced further apart [8]. However, while less frequent data collection can provide insight into the outcomes of VR interventions, they may overlook shorter-term symptom changes between assessments.

What is relatively unknown is whether frequent data collection is more informative for measuring patient-reported outcomes (PROs) than infrequent data collection. In a broader review, Peasgood et al [8] found that daily assessments tend to yield lower symptom ratings than 7-day recall, suggesting that frequency may impact PRO sensitivity. Conveniently, VR devices already allow for frequent data collection. In-device surveys can use approaches such as visual analog scales [9], asking participants how they feel in the moment to collect data as close to the VR intervention as possible with minimal recall bias [10]. Such data can be collected and stored on VR devices for retrieval when devices are returned to the research team. Alternatively, health care providers’ daily notes can be used to describe patients’ symptoms of their patients experiencing VR [11].

To the best of our knowledge, no studies have directly compared daily and weekly PRO data with a VR intervention, especially one administered at home, with head-mounted display (HMD) and desktop options, and with or without guided imagery—a therapeutic technique shown to enhance VR’s effects in some contexts [12]. This knowledge gap is especially relevant in cancer care, where patients experience fluctuating symptoms and may benefit from more tailored, flexible interventions and data collection methods [13,14].

Therefore, in this study, we examined weekly and daily data from a VR intervention on PROs among patients with varying stages of cancer diagnoses. Our intervention consisted of a virtual computer-generated natural environment similar to other virtual environments that distract and induce relaxation [15,16]. We additionally examined how adding guided imagery to our intervention influenced PROs. Guided imagery is audio narration meant to invoke one or more of the senses and guide an individual to mentally visualize experiences to reduce pain, anxiety, and depression and to improve well-being [12]. To generalize to more types of digital health interventions, we provided the intervention at 2 immersion levels—delivery in an HMD and in “desktop VR”—where patients interact with a 3D multimedia environment on a computer screen [17,18]. This investigation builds off past research showing VR and guided imagery interventions in isolation can improve PROs among patients with cancer [19-28]. Our research question was: Is collecting data at weekly intervals sufficient to predict trends in PROs, or is daily data collection more informative?

## Methods

### Study Context

This study was part of a larger project examining the impact of virtual reality–assisted guided imagery (VRAGI) dimensions on cancer pain in the home setting [29]. The original intent was to assess the efficacy and safety of VRAGI on opioid use, anxiety, depression, and fatigue and compare those effects with a VR program absent of guided imagery and laptop versions of these two interventions (with and without guided imagery). The results reported here are limited to outcomes available from data collected at both daily and weekly intervals. Only these specific data and outcomes could answer the research question for this study. The completed CONSORT (Consolidated Standards of Reporting Trials) checklist for pilot and feasibility studies [30] is provided in Checklist 1.

### Ethical Considerations

The study was approved by the Prisma Health institutional review board (Pro00114598) and published on ClinicalTrials.gov (NCT05348174). Two clinics at Prisma Health Cancer Institute in Greenville, SC (outpatient oncology and palliative care) were the sites of recruitment. This institute is accredited by the American College of Surgeons Commission and serves over 10,000 cases per year by more than 30 adult oncology specialists. Recruitment took place through flyers as well as word-of-mouth by the clinical and research staff during inpatient visits. All patients provided verbal or written informed consent, which occurred 2‐3 weeks before the intervention start date. Patients earned a US $25 e-gift card at the end of weeks 1 and 2 if they completed ≥75% of the surveys during each of those weeks, a third US $25 e-gift card if they completed ≥75% of the surveys and initiated the device return after week 3, and a fourth US $25 e-gift card if they completed ≥75% of the survey at week 6. Respondents were assigned a unique, nonidentifiable participant number, and collected data were associated with this number rather than identifiable information to maintain their confidentiality.

### Eligibility and Preliminary Screening

Verification of eligibility relied on electronic health records (EHRs) and follow-up phone conversations with the study coordinator. Inclusion criteria included patients >18 years of age, baseline pain score on the Edmonton Symptom Assessment Scale (ESAS [31]) ≥4 (mean score at 7-day screening), ability to provide consent and willingness to comply with all study procedures, and ability to comprehend spoken and written English. We initially set the inclusion criteria to also include an advanced cancer diagnosis—defined as incurable cancer, including locally advanced and metastatic cancers with no plan for resection during the study period—but broadened this criterion to a cancer diagnosis to expand the eligible sample pool. Exclusion criteria are as follows: patients >65 years of age; having a condition that interfered with VR such as a history of seizures, facial injury precluding safe placement of an HMD, or other visual or hearing impairment; previously participated in a VR clinical trial; underwent a surgical procedure within 8 weeks; diagnosed with serious mental illness; have brain metastases; have a neurocognitive disorder according to past medical history; have a prognosis of <3 months from the time of enrollment per treating oncologist; experience current substance abuse; or have experienced complex childhood trauma.

All eligible patients completed a 4-day screening, which evaluated their adherence to complete surveys. The screening survey used the same instruments as the weekly surveys in the actual study. Patients who completed fewer than 50% of the surveys provided during the screening were dismissed before the randomization process and intervention.

All but one member of the research team were blinded to the intervention group assigned to each participant. The one exception was a single researcher who conducted the randomization and device shipping and receiving. Patients were partially blinded to the treatment group. They knew the device type (laptop vs HMD) but were unaware of assignments to AGI (with guided imagery) versus NG (no guided imagery) groups.

### Intervention

Patients were randomly assigned, while considering gender, to one of four conditions: VRAGI, VR-NG (virtual reality with no guided imagery), Laptop-AGI, and Laptop-NG. VR meant the intervention was delivered via an HMD, specifically a Meta Quest 2, while the laptop meant it was delivered on a Windows laptop (13″ or 15″ screen with 1080p resolution). AGI meant the intervention included guided imagery. NG meant the content included no guided imagery and presented simply the nature-based imagery and soundscape.

All 4 conditions were experienced at the patients’ home while seated. During the study, patients were asked to engage in the intervention daily (15‐20 minutes/day) for 3 consecutive weeks. During the intervention, patients were allowed to undergo any cancer treatments outside the exclusion criteria, as prescribed by their health care provider.

All devices and accessories were packaged, shipped to patients’ homes, and ready to be used once unpacked. Virtual content was preloaded as an offline app, so no internet was needed, and patients navigated to the app with the provided instructions. Patients in the VR conditions were trained on using the HMD through a printed color manual. Patients in the laptop conditions were provided with a similar manual. To align with the audio experience of the HMD’s near-ear speaker, we provided headphones for patients in the laptop conditions to wear during the intervention. Patients could receive help from researchers by email and a telephone hotline for technical support.

### Virtual Content

**Figure 1.**
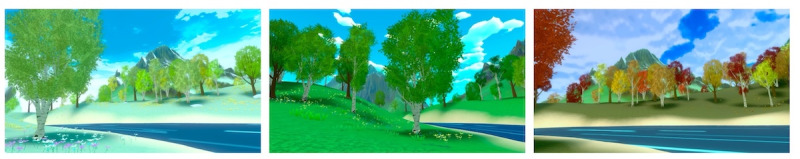
Seasons available in the virtual environments.

The virtual content consisted of a computer-generated immersive virtual landscape with nature-based imagery (ie, trees, birds, mountains, water, and birds) aligned with the landscape preferences and perceived restorative outcomes of past virtual nature scenery [16,32,33]. All conditions also had a nature-based soundscape (ie, water flowing and birds chirping). Additionally, the AGI conditions included an omnipresent vocal narration that overlapped with the imagery in the virtual scene and steered users through mental escape, redirection of attention, anxiety alleviation, and a nonjudgmental acceptance psychological process [29]. Patients were asked to complete the intervention in calendar order (ie, week 1 was the spring season, week 2 was the summer, and week 3 was the fall) (Figure 1).

The virtual content was developed across 14 months (December 2020 to February 2022) by an interdisciplinary collaboration of physicians, mental health counselors, human systems engineers, and VR content experts. Patients in all 4 conditions saw the same virtual content and could look around from all angles by moving their heads in the HMD or laptop cursor. No intervention allowed for “walking” within the virtual world.

### Measures

#### Patient Characteristics

Demographic data were provided from the patient’s EHR. These data included date of birth, age, sex, marital status, education, household income, employment status, insurance status, and place of birth. An EHR review was also used to collect patients’ medical record numbers, type of cancer, and other health status measures.

#### Weekly Data Collection of Patient-Reported Outcomes

Weekly outcomes of pain, anxiety, depression, and well-being were measured with the ESAS [31]. The ESAS is a 9-item patient-rated symptom instrument developed for use with patients receiving palliative care. In this analysis, we used the individual items measuring patient-reported anxiety, depression, pain, and well-being on an 11-point visual analog scale (ie, 0=none to 10=worst possible). The ESAS has been extensively validated for assessing symptom intensity, including among patients with cancer [34]. A change of 1 point in the ESAS has been suggested as clinically significant [35].

All patients were prompted via an email through REDCap and asked to fill out the ESAS at the start and end of each intervention week (days 1, 7, 8, 14, 15, and 21). They also received an email reminder through REDCap if they did not complete the ESAS before the end of that day. The weekly response was recorded and stored in REDCap alongside other instruments collected weekly [29].

#### Daily Data Collection of Patient-Reported Outcomes

**Figure 2.**
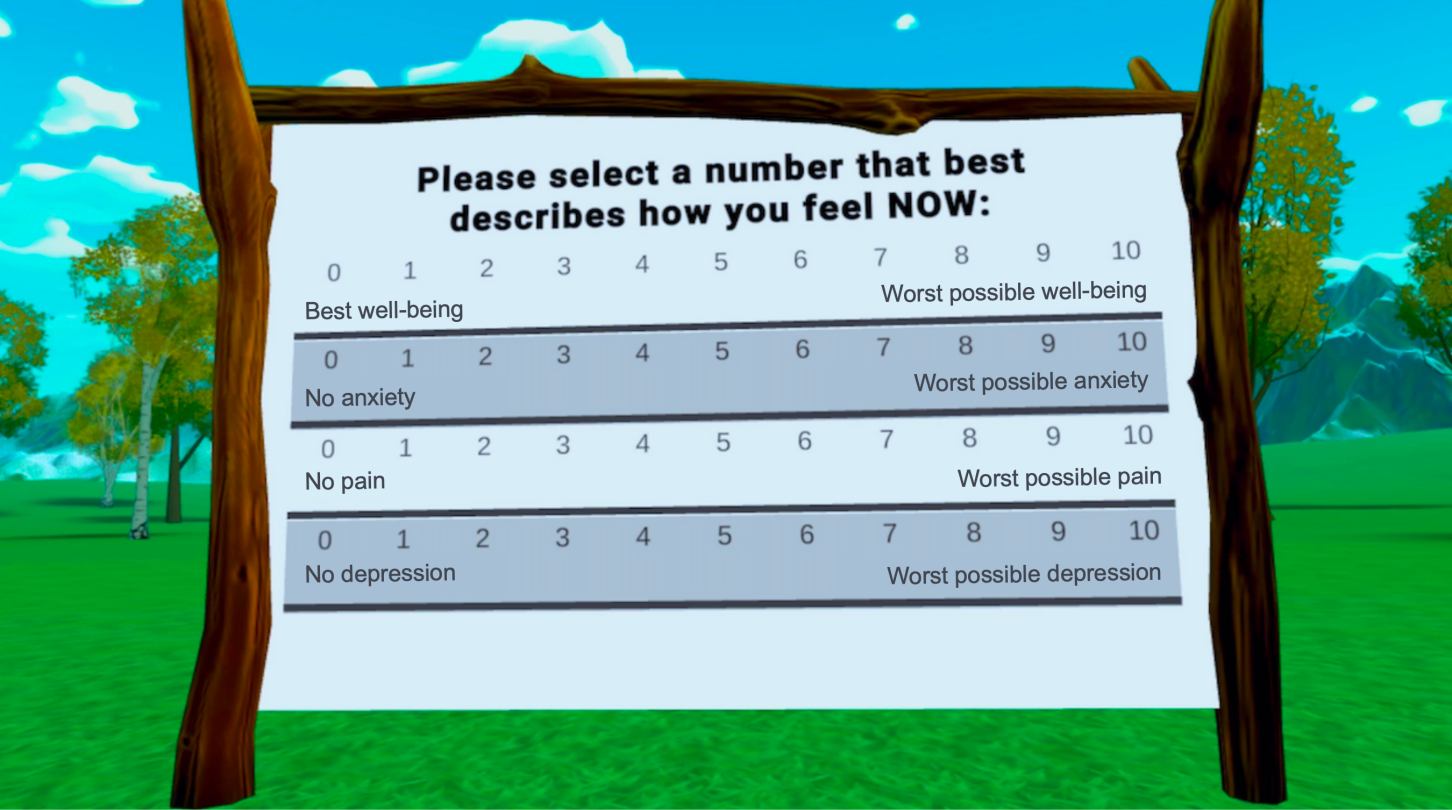
In-device survey for daily data collection of patient-reported outcomes.

Daily outcomes of pain, anxiety, depression, and well-being were measured with an in-device survey instrument designed for this study that was administered before and after each VR session on an 11-point visual analog scale (Figure 2). Patients were required to answer all the survey items before starting the VR intervention and again before quitting the VR application. The order of the survey items was randomized each time they were shown. The daily responses were recorded and stored on the study device (HMD or laptop) and retrieved by the researchers once the patients returned the device.

### Data Availability and Analysis

We faced considerable challenges in recruiting, consenting, and retaining our sample of patients with cancer. Across 17 months of recruitment (February 2023 to January 2024), we collected usable daily and weekly data from 8 patients. All members of the research team were in consensus to stop the trial due to these challenges and initiate data analysis. Due to the small sample size, this pilot study was not powered for inferential statistical analyses. Accordingly, we employed a descriptive approach using visual inspection of trends to explore preliminary patterns in PROs. For daily data, we calculated pre-post differences for each session and applied LOESS (locally estimated scatterplot smoothing) to visualize time trends by intervention group. For weekly data, raw group means were plotted without smoothing, given the limited number of time points. No imputation was performed; only complete observations were used for each data stream. Ultimately, this visual strategy of analysis was aimed at addressing our research question and generating hypotheses that could inform future research.

## Results

### Patient Recruitment and Characteristics

**Figure 3.**
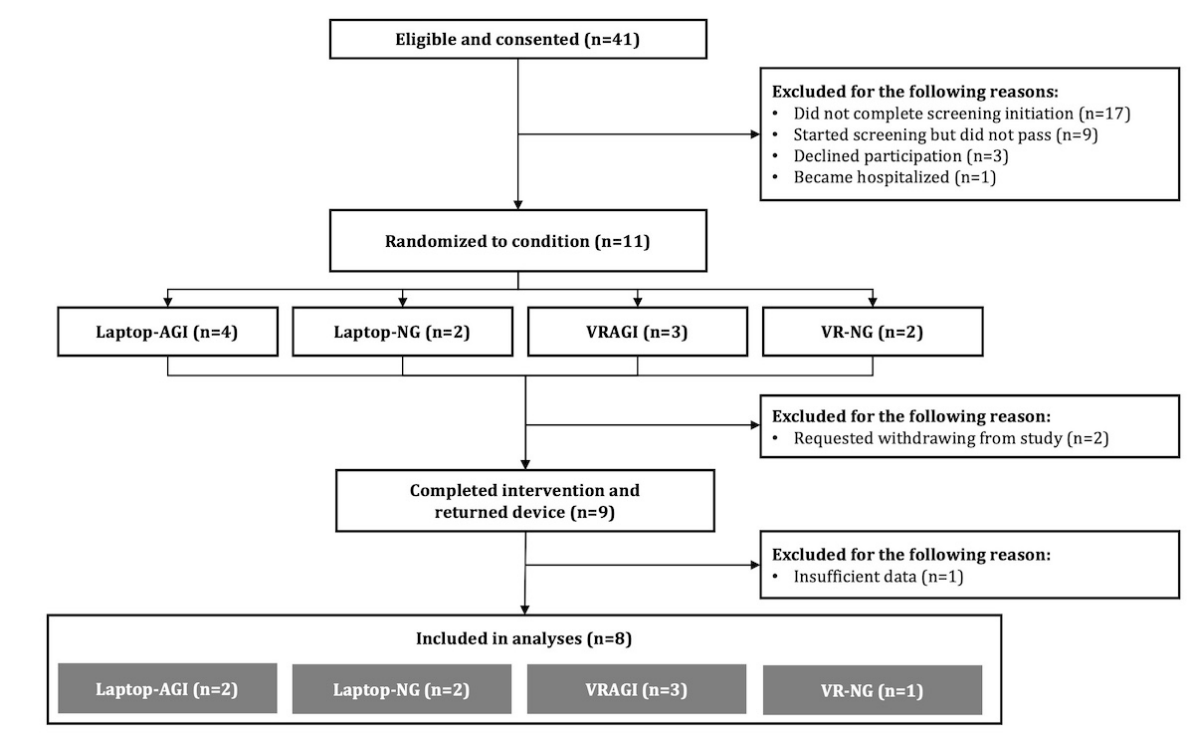
Study flowchart of patient recruitment and available data. AGI: with guided imagery; NG: no guided imagery; VRAGI: virtual reality–assisted guided imagery; VR-NG: virtual reality with no guided imagery.

We consented 41 eligible patients, but only 11 passed the screening week criteria (Figure 3). Nine patients were then randomized to VRAGI (n=3), VR-NG (n=1), Laptop-AGI (n=3), and Laptop-NG (n=2), but one patient in the Laptop-AGI condition only completed the intervention once, providing 1 day of in-device data. Therefore, our final sample size for analysis was 8.

Table 1 shows the characteristics of the patients. One-quarter were male, and the average age was 49.1 (range 30-60). All patients were non-Hispanic White. In contrast, dropout participants (n=33) had a mean age of 55 years, with 55% (n=18) female, 67% (n=22) White, 33% (n=11) African American, and 91% (n=30) non-Hispanic.

**Table 1.** Characteristics of patients.

Characteristic	Total (n=8)	Laptop-AGI^a^ (n=2)	Laptop-NG^b^ (n=2)	VRAGI^c^ (n=3)	VR-NG^d^ (n=1)
Age (years), mean (SD)	49 (10)	59 (2)	51 (8)	44 (13)	42 (0)
Sex, n (%)					
Male	2 (25)	0 (0)	1 (50)	1 (33)	0 (0)
Female	6 (75)	2 (100)	1 (50)	2 (67)	1 (100)
Race, n (%)					
White	8 (100)	2 (100)	2 (100)	3 (100)	1 (100)
Ethnicity, n (%)					
Non-Hispanic	8 (100)	2 (100)	2 (100)	3 (100)	1 (100)
Marital status, n (%)					
Single	1 (13)	0 (0)	0 (0)	0 (0)	1 (100)
Married	6 (75)	2 (100)	2 (100)	2 (67)	0 (0)
Not reported	1 (13)	0 (0)	0 (0)	1 (33)	0 (0)

a AGI: with guided imagery.

b NG: no guided imagery.

c VRAGI: virtual reality–assisted guided imagery.

d VR-NG: virtual reality with no guided imagery.

### Weekly Versus Daily Data on Pain

**Figure 4.**
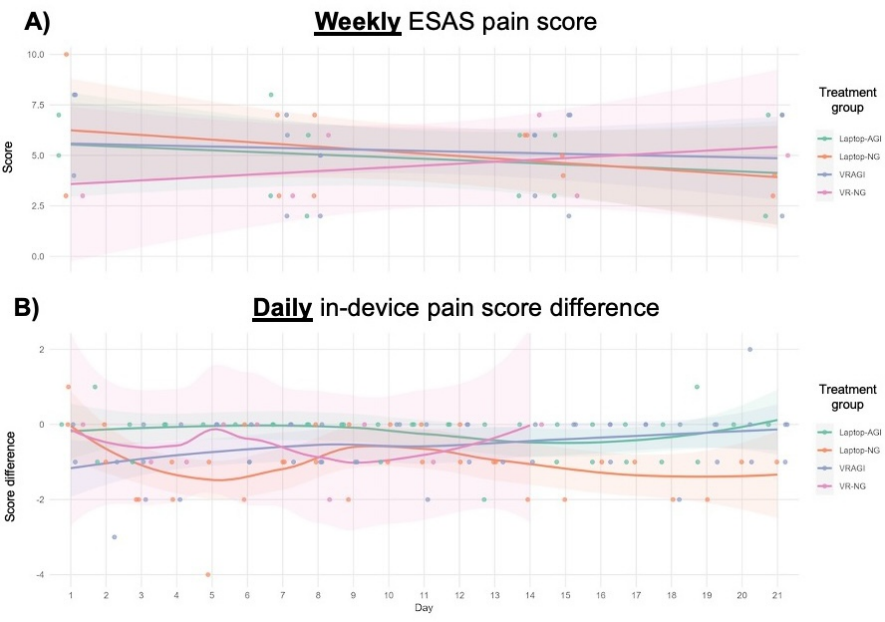
Changes in pain scores based on data collected at weekly versus daily intervals. (**A**) Weekly ESAS pain scores. (**B**) Daily in-device pain score differences. AGI: with guided imagery; ESAS: Edmonton Symptom Assessment Scale; NG: no guided imagery; VRAGI: virtual reality–assisted guided imagery; VR-NG: virtual reality with no guided imagery.

Weekly pain score data showed no consistent patterns (Figure 4). In contrast, daily data from the Laptop-NG and VR-NG conditions revealed double-bottom patterns (ie, initial decline, recovery, and secondary decline). Patients reported improvements in pain within the first few days of the intervention and again 1-2 weeks afterward. While these results showed differences in PROs between conditions with and without guided imagery, there was insufficient evidence to suggest that the addition of guided imagery improved PROs.

### Weekly Versus Daily Data on Anxiety

**Figure 5.**
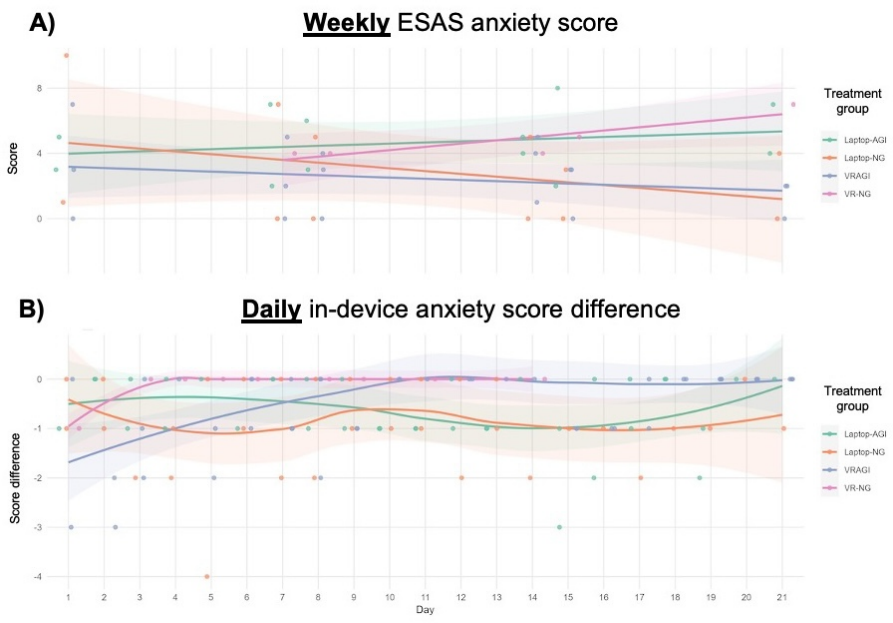
Changes in anxiety scores based on data collected at weekly versus daily intervals. (**A**) Weekly ESAS pain scores. (**B**) Daily in-device pain score differences. AGI: with guided imagery; ESAS: Edmonton Symptom Assessment Scale; NG: no guided imagery; VRAGI: virtual reality–assisted guided imagery; VR-NG: virtual reality with no guided imagery.

Weekly data from the intervention showed no strong effects on anxiety (Figure 5). The Laptop-NG condition exhibited the greatest decrease in anxiety over time, while the VR-NG condition showed the greatest increase, though both effects were modest. In contrast, daily data revealed clearer trends for the VR-NG and VRAGI conditions, with anxiety levels plateauing over time. Initial benefits were observed within the first 2 days of the VR-NG intervention and within the first week of VRAGI. However, these effects did not continue to increase with prolonged use of the intervention. These results also provided insufficient evidence that the addition of guided imagery assisted with PROs.

### Weekly Versus Daily Data on Depression

**Figure 6.**
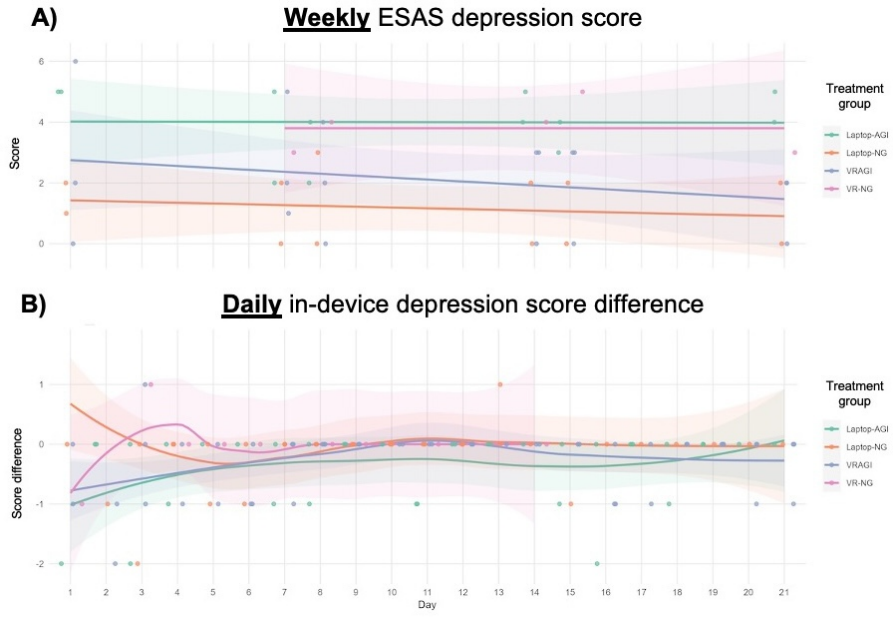
Changes in depression scores based on data collected at weekly versus daily intervals. (**A**) Weekly ESAS pain scores. (**B**) Daily in-device pain score differences. AGI: with guided imagery; ESAS: Edmonton Symptom Assessment Scale; NG: no guided imagery; VRAGI: virtual reality–assisted guided imagery; VR-NG: virtual reality with no guided imagery.

Weekly data from the intervention showed no strong effects on depression (Figure 6). However, daily data from VR-NG showed a plateauing pattern where the intervention’s benefits were observed in the first 2 days of device use but not afterward. These results once again provided no evidence that the addition of guided imagery assisted with PROs.

### Weekly Versus Daily Data on Well-Being

**Figure 7.**
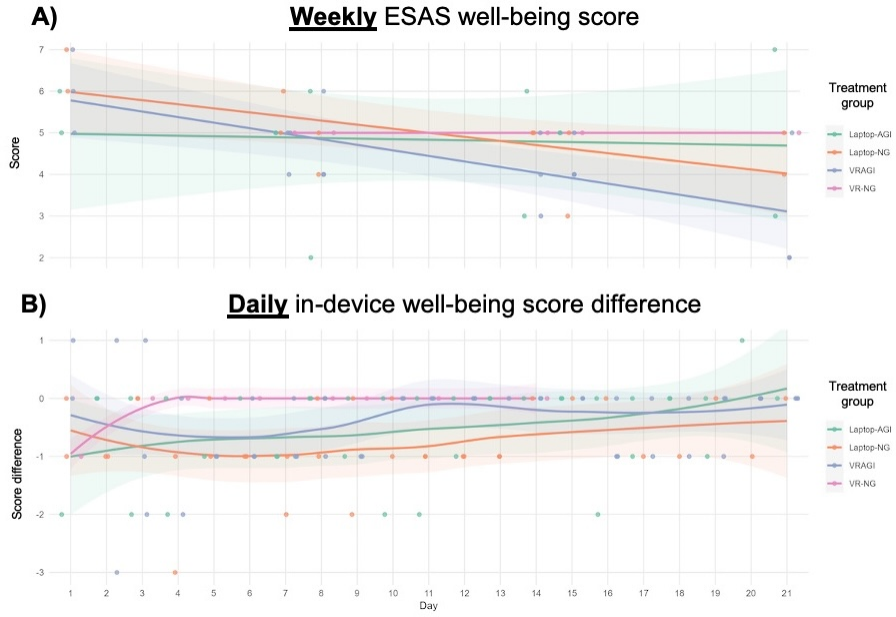
Changes in well-being scores based on data collected at weekly versus daily intervals. (**A**) Weekly ESAS pain scores. (**B**) Daily in-device pain score differences. AGI: with guided imagery; ESAS: Edmonton Symptom Assessment Scale; NG: no guided imagery; VRAGI: virtual reality–assisted guided imagery; VR-NG: virtual reality with no guided imagery.

Weekly data from well-being scores suggested downward trends for VRAGI and VR-NG (Figure 7). Daily data from VR-NG showed a plateauing pattern similar to the depression data pattern, where the intervention’s benefits were observed in the first couple of days but not afterward.

These results provided evidence that the addition of guided imagery may have assisted with PROs measured weekly but not daily—results which may inform future trials. No adverse events or harms were reported.

## Discussion

### Summary of Main Findings

In summary, 8 patients analyzed within this technology-based intervention provided PRO data on daily and weekly schedules. Weekly data showed inconsistent patterns, while daily data revealed more informative trends such as double-bottom patterns and plateauing effects for some outcomes and conditions.

Weekly data for pain, anxiety, and depression PROs had no consistent patterns over time. However, weekly data suggested that the laptop intervention with virtual nature imagery (and no guided imagery) had the greatest decrease in anxiety. The VR HMD intervention with virtual nature imagery (and again, no guided imagery) showed the greatest increase in anxiety over time.

Daily data showed different findings. The greatest benefit for pain was observed in the laptop and HMD interventions (without guided imagery) but only within the first few days and then 1-2 weeks later. Daily data also showed benefits for anxiety among the HMD conditions but only within the first few days to 1 week. Likewise, depression and well-being plateaued after a few days in the VR condition without guided imagery.

Comparing weekly and daily data illustrates the importance of PROs at varying time points during a digital intervention. Without daily data, for instance, there may be fewer insights into the trends and patterns over the course of the intervention.

The rationale behind combining VR and guided imagery lies in complementary mechanisms for modulating attention and emotional processing. Nature-based VR can benefit patients through distraction and relaxation since natural landscapes can capture people’s soft fascination, align with our evolutionary needs of survival, and recall positive memories [15,16,33]. Meanwhile, guided imagery can redirect the user’s attention away from the negative emotional processing of pain [12]. While our results did not confirm these additive effects of nature-based VR and guided imagery, further work with fully powered samples is warranted to disentangle whether these 2 modalities complement each other and what mechanisms drive therapeutic benefits.

### Implications for Researchers and Clinicians

This study has implications for researchers and clinicians interested in using VR for improving PROs. It is important to consider when designing a VR clinical trial how frequently to measure PROs. There are benefits and drawbacks to daily versus weekly survey administrations, such as patient burden or biases resulting from repeated survey instruments. Still, daily surveys may allow for in-depth tracking of an intervention’s efficacy. Daily approaches may thus allow for a more personalized approach to these interventions. Research coordinators could communicate with patients after PROs severely decrease, increase, or plateau and offer intervention support or follow-up data collection on the severe changes in PROs.

Daily PRO collection may also allow researchers and clinicians to track when a patient group or individual may discontinue an intervention. For example, a patient group may be on a standardized intervention schedule (ie, 8 weeks), but their daily PROs may plateau in week 4. This indicates that patients may not need to continue the intervention, saving resources and time and ultimately helping patients reap the benefits of PROs quicker and through a less demanding or intensive intervention process. As a hypothetical example, an adaptive clinical trial with cancer patients may begin with daily VR sessions and complete short in-device PROs after each session. If a participant reports sustained symptom relief (eg, ≥1-point ESAS improvement maintained for 3 consecutive days), the intervention could be reduced in frequency to every other day. Alternatively, if a patient reports worsening symptoms or stagnation, the device could trigger the researchers to provide the patient with additional support (eg, switching VR modalities or initiating a follow-up call) or terminate the intervention. Still, it should be acknowledged that plateauing of daily PRO surveys could also be due to familiarity of survey items, as patients may have defaulted to providing the same values each time they saw the in-device surveys, even when items are randomized.

Patient samples provide opportunities for applied research where VR and VR interventions benefit those in greatest need. However, we experienced challenges when using patients, specifically those with cancer. Previous literature emphasizes several barriers to participation, including fluctuating symptom burdens, fatigue, logistical issues, and psychological stress associated with illness progression, which collectively complicate sustained involvement in clinical research [36]. In our study, among the 41 patients who provided informed consent, we observed substantial attrition before the intervention initiation, with 80% (33/41) not choosing to follow up, including all 11 patients who were Black—a disproportionate dropout raising concerns about equity and suggesting that our recruitment and screening may have inadvertently failed to support the inclusion of underrepresented populations. This high attrition among Black participants aligns with a broader historical pattern, rooted in longstanding systemic mistrust stemming from unethical research practices, which have understandably led many Black Americans to be cautious about participating in clinical research [37]. Integrating trusted health care providers early in the recruitment and retention process, as well as culturally tailored communication strategies, may have helped mitigate these issues and improve retention rates [38,39].

More broadly, this relatively high dropout rate during screening may be attributed to multiple factors. For instance, the extended timeline between consent and screening (1 week) and the involvement of non–health care personnel (the screening-intervention process was supervised by nonclinical research team members) may have introduced communication inefficiencies and spurred dropout. Streamlining recruitment-to-screening processes and integrating health care team members in screening processes might improve early retention rates. Embedding trusted health care providers as contacts for VR interventions may also ensure patients are invested throughout the study process. Trusted health care providers are likely positioned to build trust and address concerns throughout the study process. This trust-based approach may be crucial in studies requiring sustained patient engagement, such as daily interventions.

Despite the high preintervention attrition, the retention rate among patients who commenced the intervention was encouraging, with 73% (8/11) of patients completing the study protocol. Patients who demonstrated higher engagement and motivation during the screening process tended to show better adherence during the intervention. This suggests that VR interventions measuring PROs may benefit from screening processes that mimic the surveys and patient responsibilities required during the intervention.

For researchers and clinicians using at-home VR interventions, there are challenges to balancing device functionality and monitoring. We ran into minor incidents, including unauthorized device use by a minor, concerns about illegal data entry, devices malfunctioning and needing to be replaced, and packages missing before arrival. Still, we expect our challenges to have been greater if devices were connected to the internet. Future protocols can be designed to assist VR researchers and clinicians to balance competing priorities, including stable device performance, patient safety, sensitive information, and unauthorized use, while still enabling data collection of PROs and adherence.

### Strengths and Limitations

This study had both strengths and limitations. One strength was the combination of immersive nature imagery in VR with guided imagery that could be self-administered at home without an internet connection. The completion rate among our intervention initiators was high (>70%), suggesting this intervention was generally feasible once patients completed the consent and screening criteria. However, our findings were further limited by the small sample size given significant recruitment constraints and our inability to conduct live monitoring or biofeedback to track physiological indicators and validate PROs.

Further limiting our study were the methodological differences between the daily and weekly survey measures, given that the original study was not designed for this purpose. Patients were required to complete daily surveys to access the intervention, whereas weekly surveys were optional and incentivized. These differences in requirements, incentives, and reminder frequencies between daily and weekly data collection methods could have influenced participant response patterns and introduced biases, complicating direct comparisons of the datasets. Still, our exploratory findings may be informative for several reasons. First, patterns emerged in the daily data—such as plateau effects—which weekly data failed to detect, highlighting the inherent sensitivity of more frequent assessments. Second, our findings align with other studies suggesting that daily assessments may more accurately capture transient symptom changes [8]. Finally, recognizing such nuances from our exploratory data can inform future study designs, emphasizing the need to consider data collection frequency carefully to balance the sensitivity and respondent burden of PROs.

### Conclusions

When designing VR interventions, it may be important to consider how often to collect PROs. This pilot study suggests that daily data collection may be more informative for researchers and clinical teams than less frequent data collection. Future research is needed to substantiate these results—which were not only mixed but also based on a small sample size—and to evaluate how data collection frequency and VR interventions such as guided imagery impact PROs.

## Supplementary material

10.2196/73506 Checklist 1 CONSORT-eHEALTH checklist (v1.6.1).

## Abbreviations

AGI: with guided imagery
CONSORT: Consolidated Standards of Reporting Trials
EHR: electronic health record
ESAS: Edmonton Symptom Assessment Scale
HIPAA: Health Insurance Portability and Accountability Act
HMD: head-mounted display
LOESS: locally estimated scatterplot smoothing
NG: no guided imagery
PRO: patient-reported outcome
REDCap: Research Electronic Data Capture
VR: virtual reality
VR-NG: virtual reality with no guided imagery
VRAGI: virtual reality–assisted guided imagery
